# Hydrocolpos in a mixed-breed dog: a case report

**DOI:** 10.3389/fvets.2026.1910776

**Published:** 2026-09-03

**Authors:** Christoph Leber, Thomas Rohwedder, Sina Rehbein-Manchi

**Affiliations:** Valera Small Animal Clinic, Berlin, Germany

**Keywords:** canine congenital sexual disorders, dog, episiotomy, hydrocolpos, traumatic induced vaginal atresia, vaginal atresia, vaginal polyp

## Abstract

A 3.5-year-old, 5.2-kg mixed-breed dog was presented with persistent stranguria after being neutered due to dystocia 6 months ago. Plain abdominal radiographs revealed an intrapelvic soft-tissue attenuating mass that displaced the urinary bladder cranially and constricted the descending colon. Abdominal ultrasonography yielded inconclusive results. Computed tomography revealed the mass as a tubular, hypodense structure compressing the urethra and the descending colon. This finding was defined as hydrocolpos induced by an imperforate hymen, segmental vaginal aplasia, or a vestibulovaginal disorder. Vaginoscopy revealed vaginal atresia and a stem-shaped mass obscuring the ostium urethrae externum. Retrograde cystography showed a significantly dilated urethra behind the obscured ostium urethrae externum. A pedunculated mass (polyp) was suspected to hinder the urinary outflow. A minimal invasive resection of the polyp and widening of the urethra were performed. Normal micturition was observed for 2 months. In the 9th week, the patient returned with tenesmus. An invasive procedure involving episiotomy, evacuation of the hydrocolpos, and creation of a stoma between the mucous membrane of the hydrocolpos and the vaginal mucosa led to permanent resolution of the clinical signs. Only a few case reports exist regarding congenital hydrocolpos. As the history indicates, a traumatic origin is considered likely; therefore, we assume that this is the first case report of secondary, trauma-induced vaginal atresia, and the resulting hydrocolpos.

## Background

Canine congenital disorders have been estimated to occur in up to 6.95% of newborn dogs (1–3). Various environmental factors have a direct impact on developmental malformations during embryogenesis, including genetic factors, drugs, heavy metals, toxins, infections, and malnutrition. Various dog breeds can be affected (2). Overall, three types of abnormalities can be distinguished: chromosomal, gonadal, and phenotypic sex abnormalities. Each of these can cause various phenotypical alterations (4).

During embryonic development, the Müllerian ducts develop into the fallopian tubes, uterus, cervix, and the caudal section of the uterovaginal canal. The resulting cervix forms the junction between the uterus and vagina. The vestibule of the vagina develops from the urogenital sinus. The hymen is formed by connecting the caudal end of the vaginal canal and the vestibule. In dogs, this epithelial separation usually disappears at birth (5–7).

An imperforate hymen can occur as a dorsoventral membrane or as a circular stricture. When the vagina is completely closed by the hymen, this condition is referred to as hymenal atresia. This can lead to the accumulation of fluid due to uterine and vaginal secretions, resulting in hydrocolpos. Any error that occur during embryonic development in this area can lead to various abnormalities in the distal reproductive tract (6, 8–12).

Other congenital abnormalities of these areas include vaginal aplasia, vaginal septa, and vestibulovaginal strictures. In addition to asymptomatic cases, abnormalities of the genital tract can cause very different symptoms, such as recurrent urinary tract infections, urinary incontinence, vaginitis, vaginal discharge, infertility, inability to reproduce naturally, and dystocia. The described symptoms of hydrocolpos include dysuria, stranguria, pollakiuria, tenesmus, and abdominal pain (6, 7, 11–15).

The aim of this case report was to describe the clinical presentation, imaging findings, surgical interventions, and outcomes in a dog with suspected trauma-induced secondary vaginal atresia and the resulting hydrocolpos.

## Case presentation

A 3.5-year-old, 5.2-kg, neutered female Chihuahua mix was presented to the Valera Clinic for Small Animals, Berlin, due to stranguria. The dog had originally been rescued by an animal welfare organization in Hungary. Due to dystocia, the dog was neutered 6 months previously. Since arriving in Germany 6 weeks before presentation, she had exhibited urinary dribbling and stranguria. Whether and when these clinical signs had already occurred in Hungary is unknown. Previous treatment with antibiotics (unknown substance) and analgesics (unknown substance) had no effect.

**Figure 1:**
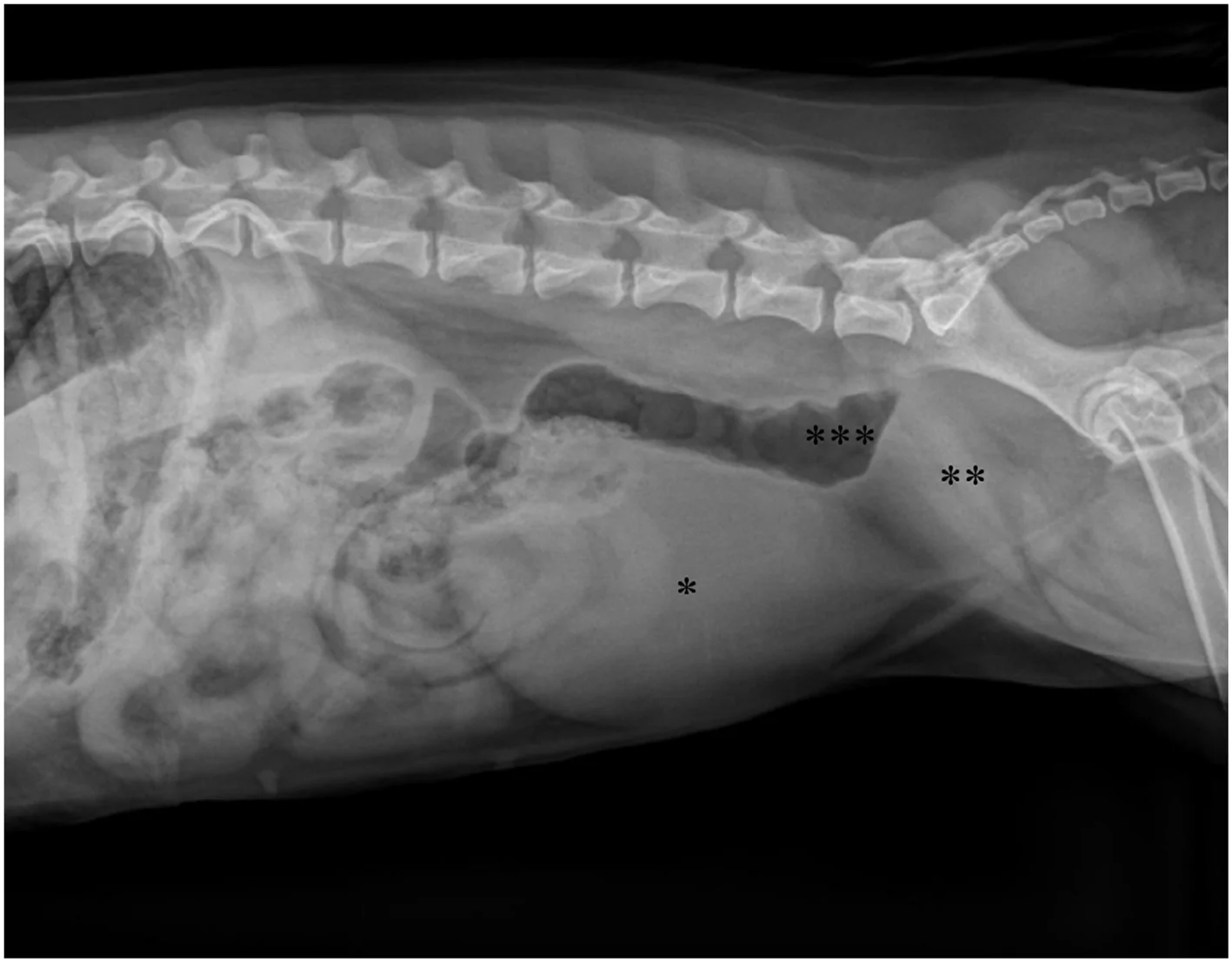
Abdominal radiograph, left lateral-lateral view: an intrapelvic mass of soft-tissue attenuation**, displacing the urinary bladder*, cranially and constricting the descending colon***.

Apart from increased abdominal tension, the general clinical examination was unremarkable. All blood parameters were within the reference range. Abdominal radiographs revealed a cranially displaced urinary bladder and constriction of the descending colon due to an intrapelvic soft-tissue attenuating mass (Figure 1).

**Figure 2:**
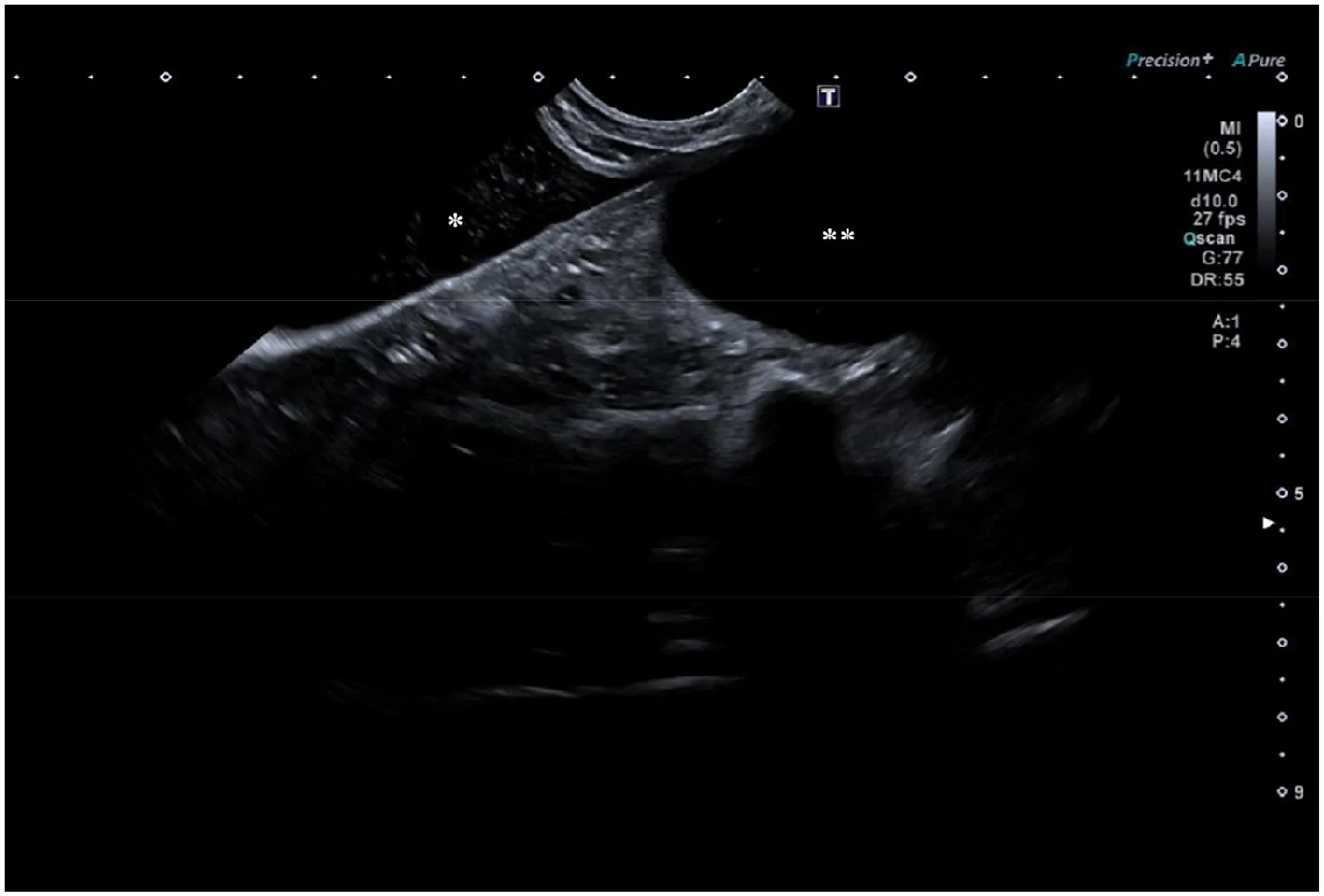
Abdominal ultrasonography: the urinary bladder with free-floating corpuscular content*; caudal to the urinary bladder, a well-marginated, round, anechoic intrapelvic structure is visible, compressing the urethra**.

Abdominal ultrasonography revealed a well-marginated, anechoic structure located caudodorsal to the urinary bladder. This structure could not be assigned to a specific organ. The urinary bladder itself contained free-floating, corpuscular components (Figure 2).

As a diagnostic and initial therapeutic step, a urinary catheter was placed. The size of the ostium urethrae externum was heavily reduced (Ø 1 mm). Urine analysis revealed active sediment containing leukocytes and erythrocytes, with suspected bacterial presence. Subsequent microbiological examination confirmed the presence of multidrug-resistant bacteria.

Fine-needle aspiration of the well-marginated, fluid-filled structure revealed a transudate, with no hints of malignancy.

**Figure 3:**
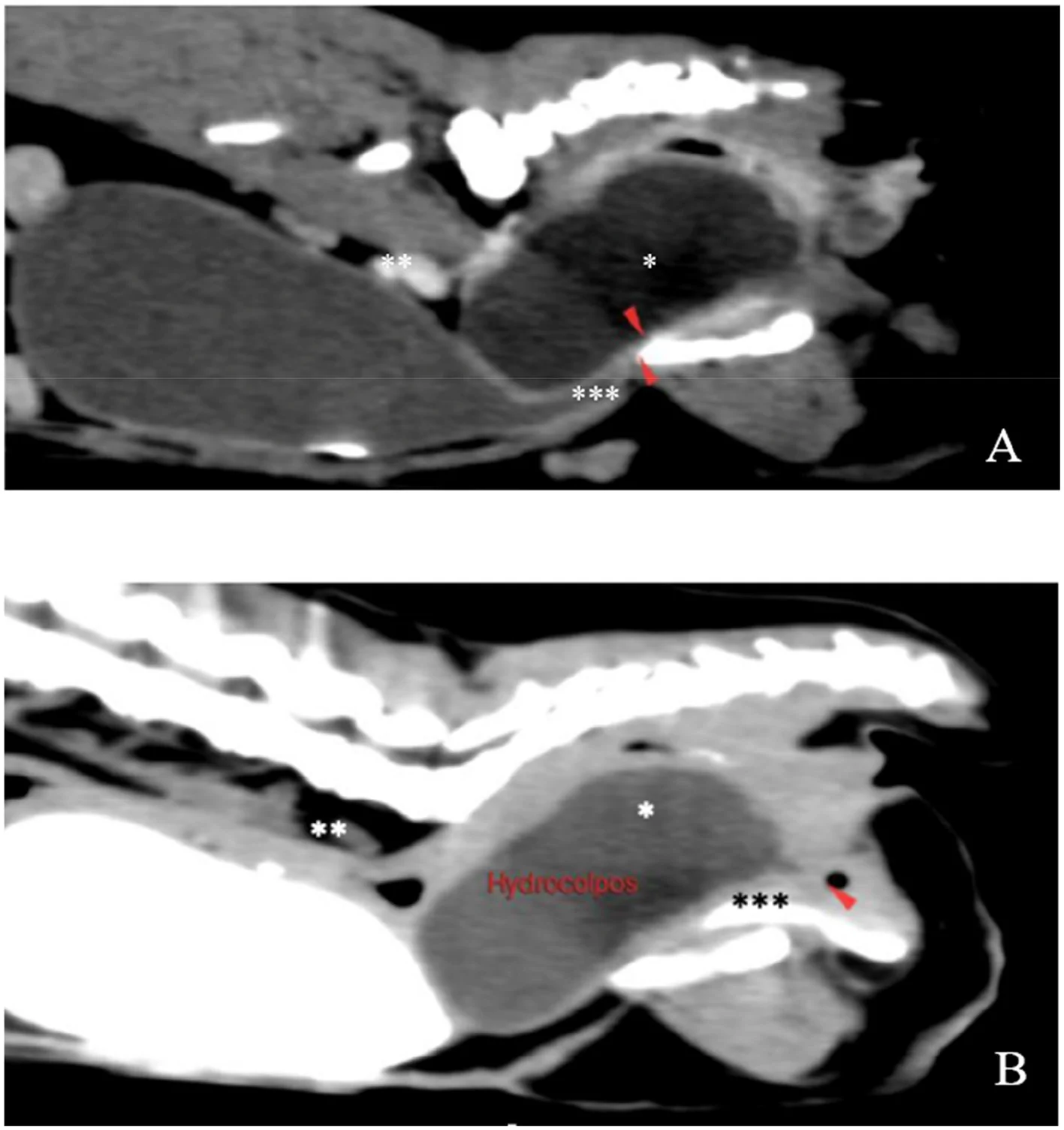
**(A, B**) Computed tomography, sagittal view: a tubular, hypodense structure^*^ between the rectum^**^ and urethra^***^, without contrast enhancement, causing compression of the urethra (two arrowheads), and an imperforate hymen (single arrowhead). Diagnosis: hydrocolpos.

Under general anesthesia (premedication: methadone 0.2 mg/kg IV (Insistor 10 mg/ml, methadone hydrochloride, Chanelle Pharma) and dexmedetomidine 2 mcg/kg IV (Dexdomitor 0.5 mg/ml, dexmedetomidine hydrochloride, Vetoquinol GmbH); induction: propofol IV (Proposure, Propofol, Le Vet Beheer B.V., the Netherlands); and maintenance: isofluran inhalation (Isofluran CP 1 ml/ml, Isofluran, CP-Pharma Handelsgesellschaft mbH), computed tomography was performed. Hydrocolpos was diagnosed (Figure 3).

**Figure 4:**
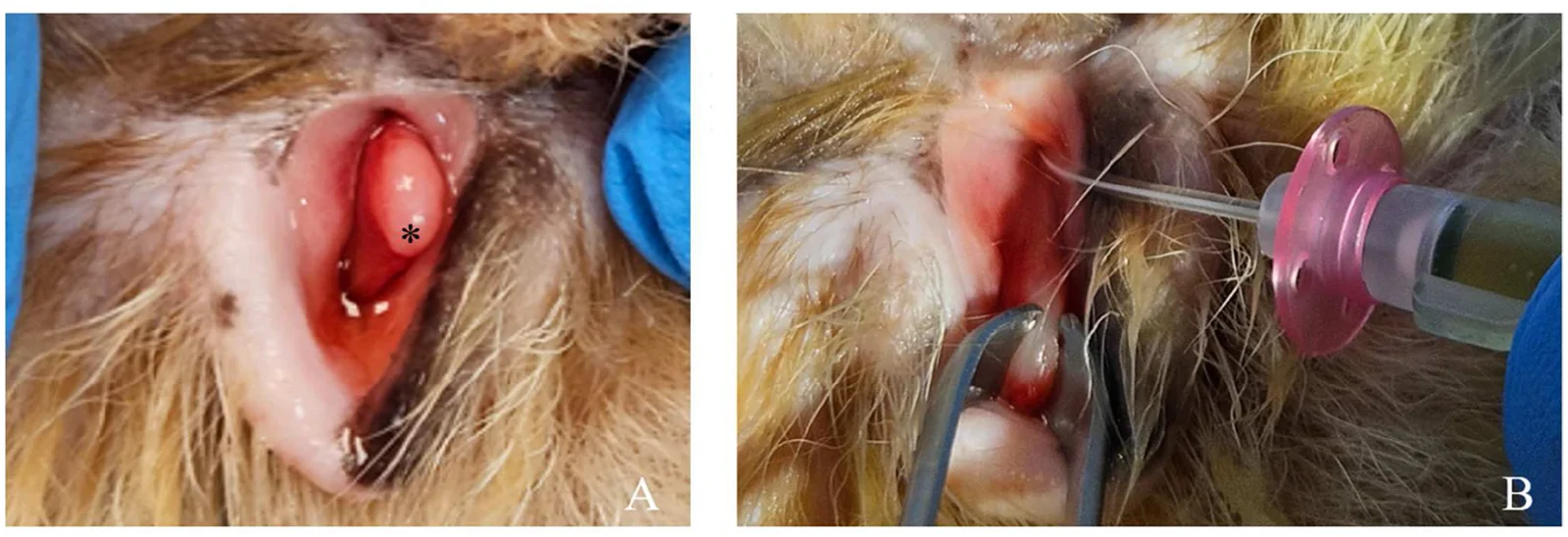
**(A, B)** Pedunculated mass^*^ with visualization of the ostium urethrae externum using a catheter of Ø 1 mm.

Vaginoscopy revealed that the labia appeared normal, but the vestibule of the vagina was absent, and the diameter of the vestibulovaginal junction was reduced, consistent with vaginal atresia. The ostium urethrae externum was anatomically in the correct position, but it was obscured by a stem-shaped mass (Figure 4).

**Figure 5:**
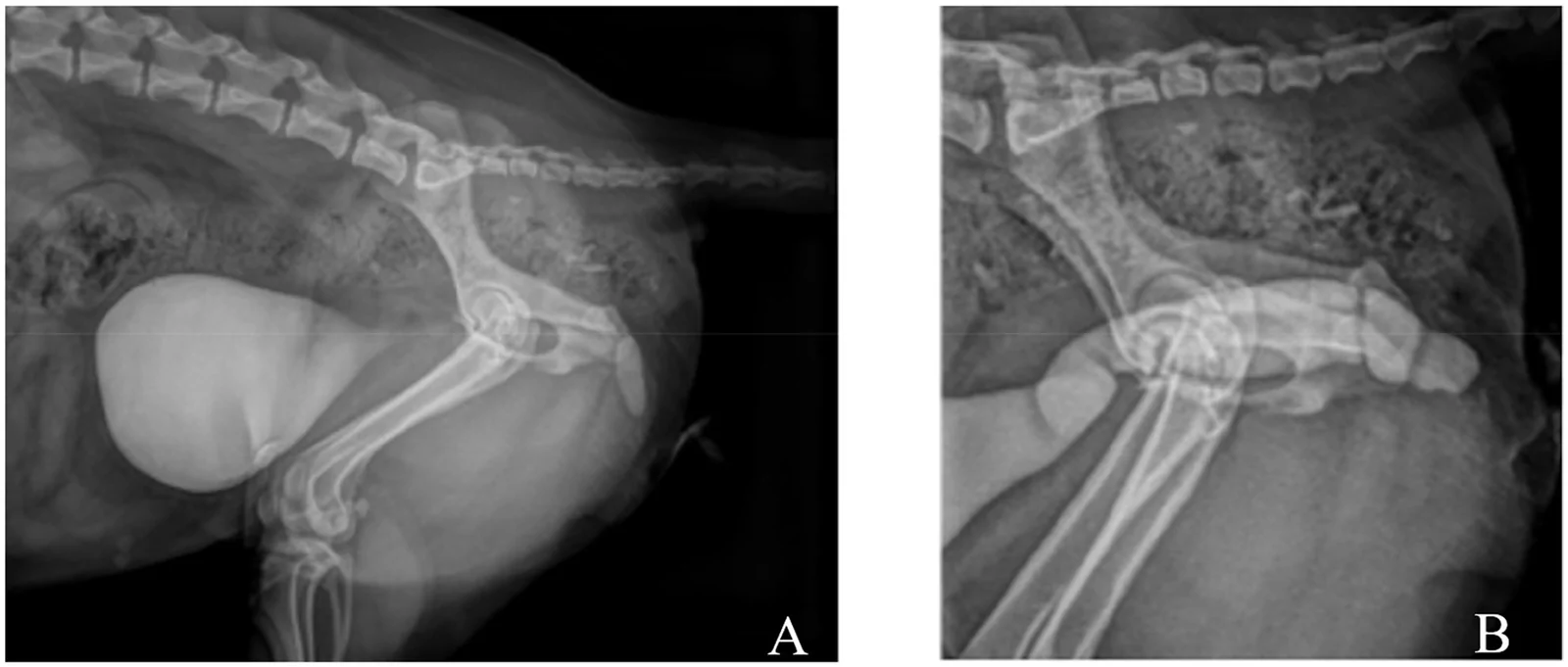
**(A, B**) Radiograph, left lateral-lateral view: 30 ml of contrast agent (1:1 diluted Xenetix 300 (Iobitridol 300 mg Jod/ml, Guerbet) was administered via a urinary catheter (Ø 1 mm). The urinary bladder was unremarkable. The urethra was significantly dilated.

**Figure 6:**
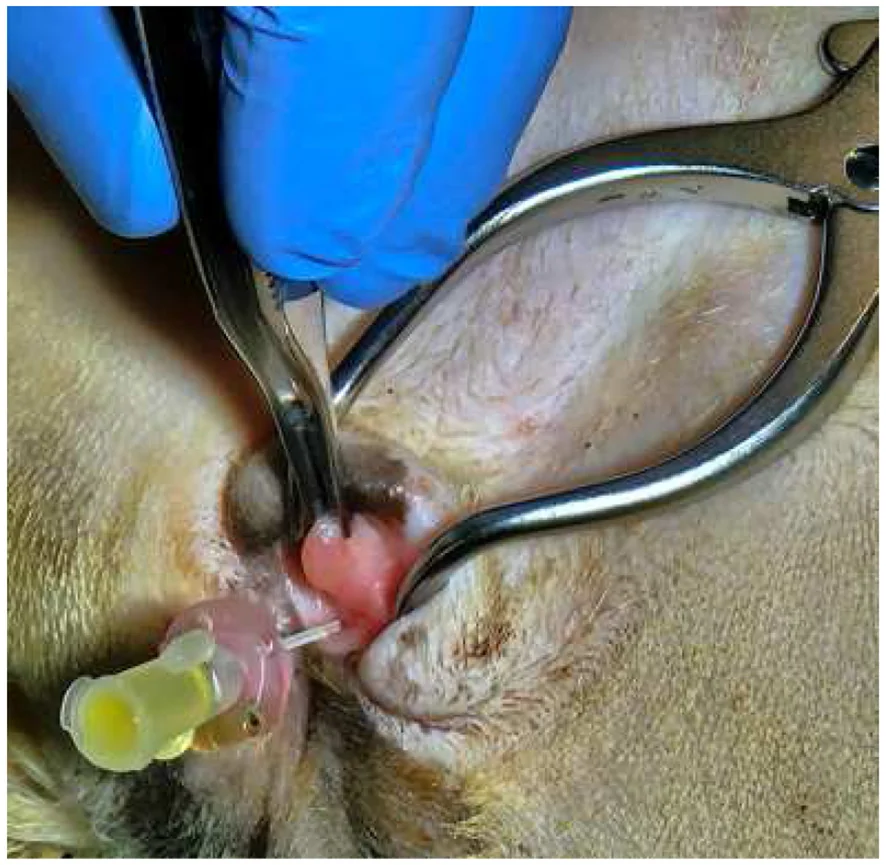
Removal of the pedunculated mass with visualization of the ostium urethrae externum using a 1-mm catheter.

A new urinary catheter (Ø 1 mm) was placed, and 30 ml of 1:1 diluted Xenetix 300 (Iobitridol 300 mg Jod/ml, Guerbet) was instilled retrogradely under high pressure to evaluate the presence of a urethral stricture. The urethra was significantly dilated behind the ostium urethrae externum (Figure 5). It was suspected that this pedunculated mass was obstructing urinary outflow and was therefore removed using electrosurgical tweezers (Figure 6).

The ostium urethrae externum was widened under endoscopic control, and a 12-French Foley catheter was inserted. The dorsal vulvar margin was freshened and apposed to the urethral mucosa using simple interrupted sutures. Histopathological analysis revealed a fibrotic polyp with mild mucosal hyperplasia.

Postoperatively, the patient received a constant rate infusion of Sterofundin ISO IV (40 ml/kg/24 h; B. Braun Vet Care GmbH), meloxicam (0.1 mg/kg PO once daily for 7 days; Melosus 1.5 mg/ml, meloxicam, Cp-Pharma Handelsgesellschaft GmbH), and nitrofurantoin (5 mg/kg PO twice daily for 14 days; Nifurantin 50 mg, nitrofurantoin, Apogepha Arzneimittel GmbH) according to the antibiogram. The catheter was removed 4 days postoperatively. Normal micturition was observed for 2 months. No wound-healing complications occurred, as confirmed by vaginoscopy.

In the 9th week, tenesmus developed, which primarily affected defecation and worsened significantly within 1 week. The perianal area was swollen, and a rectal prolapse was present. Rectal examination revealed a fluctuant mass, which was suspected to correspond to the initially diagnosed hydrocolpos. A second transabdominal ultrasonographic examination did not show any other changes compared to the previous examinations. Thus, repeat computed tomography was deemed unnecessary. Instead, the first computed tomography scan was re-evaluated before surgical intervention.

To determine whether any additional concomitant tissue alterations, which could have been missed on diagnostic imaging, were present and to attempt primary surgical resection of the hydrocolpos, a diagnostic laparotomy was chosen over episiotomy.

The previously described anesthetic protocol was combined with epidural anesthesia (mepivacaine 4 mg/kg; Intra-Epicain, mepivacaine hydrochloride, Dechra Veterinary Products Deutschland GmbH, and two boluses of fentanyl IV; 2 mcg/kg per bolus; Fentadon 50 mcg/ml, fentanyl citrate, Dechra Veterinary Products Deutschland GmbH). Amoxicillin/clavulanic acid (20 mg/kg IV; AmoxClav HEXAL^®^ 500/100 mg, Hexal AG) was administered preoperatively and every 90 min throughout the surgery. Phased hypotension was treated with dopamine (5 mg/kg/min IV; Dopamin Fresenius 50 mg/5 ml, dopamine hydrochloride, Fresenius Kabi Deutschland GmbH).

The urinary bladder was adherent to the omentum. The hydrocolpos could not be resected via laparotomy. After layer-appropriate wound closure of the abdominal incision, an episiotomy was performed.

**Figure 7:**
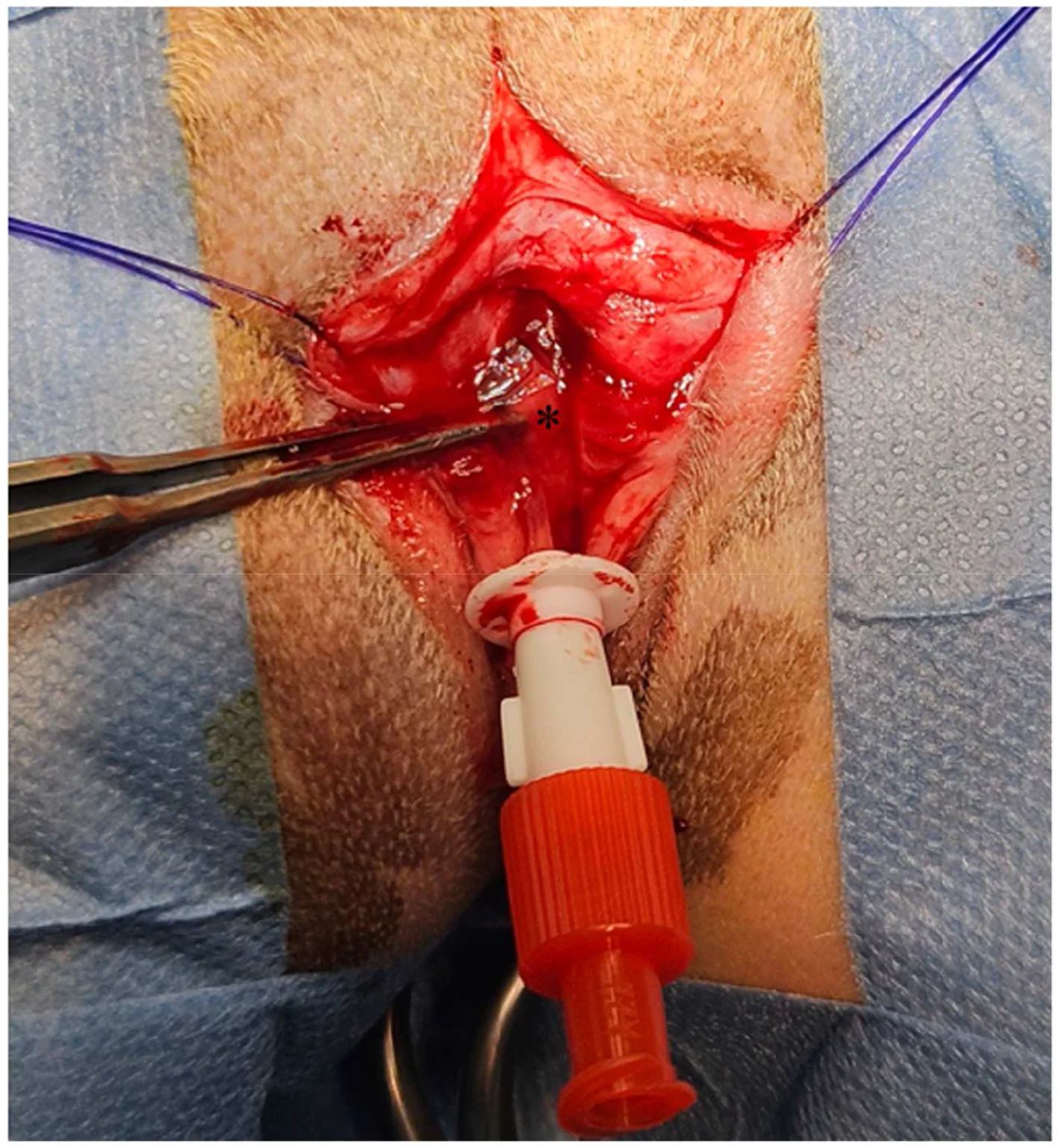
Episiotomy: insertion of a 1-mm urinary catheter, and visualization of the urethra. ^*^Hydrocolpos.

The urethral opening was visualized, and a urinary catheter (Ø 1 mm) was inserted without resistance. The hydrocolpos presented as a firm, elastic protrusion of the mucous membrane located craniodorsal to the urethra (Figure 7).

**Figure 8:**
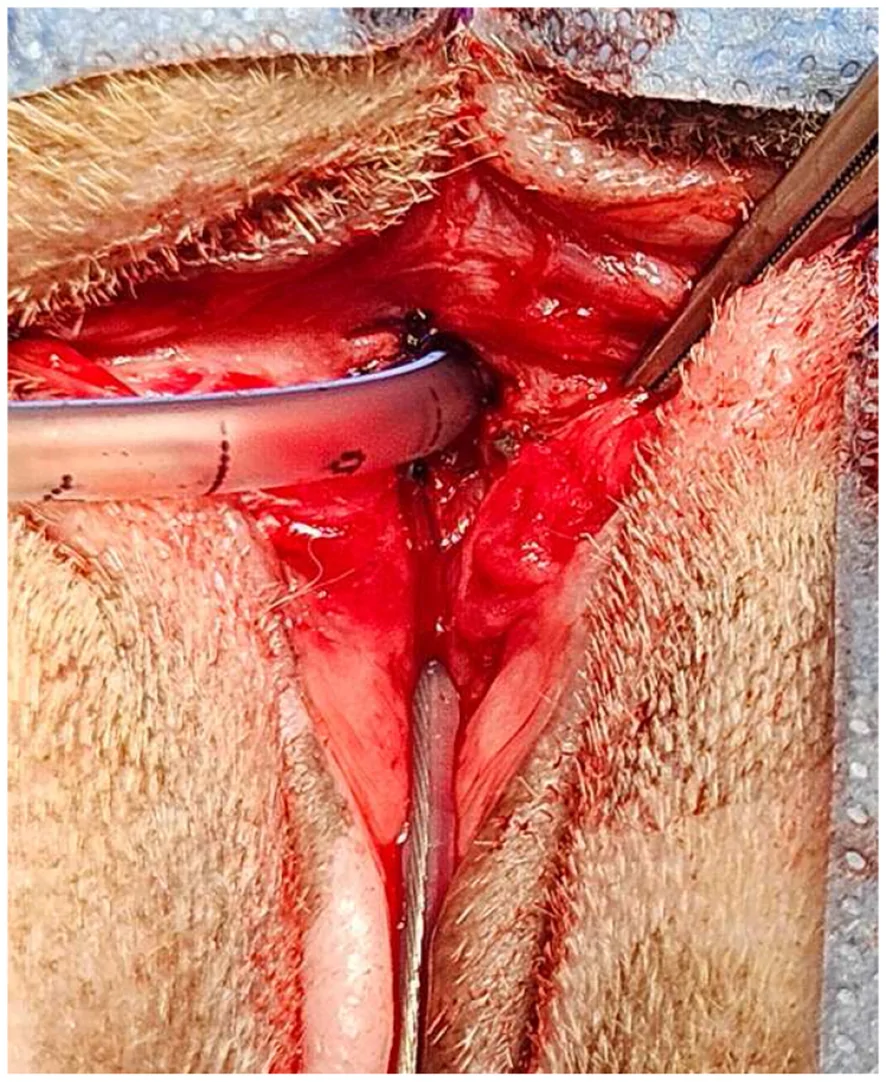
Episiotomy: insertion of a 12-French Foley catheter after widening the stoma between the mucous membrane of the hydrocolpos and the vaginal mucosa.

The hydrocolpos was opened by puncture incision using a 11-scalpel blade. A large amount of mucopurulent fluid was evacuated. A microbiological swab sample was collected. Afterward, the opened part was rinsed with sterile Ringer's lactate solution. The puncture incision was widened, and after inserting a 12-French Foley catheter, a 5-mm stoma was created between the hydrocolpos membrane and the vaginal mucosa using single sutures with Monosyn (4-0) (Figure 8). A layer-appropriate wound closure of the episiotomy incision was then performed.

Postoperatively, the patient received intravenous infusion therapy with a crystalloid solution supplemented with fentanyl (Fentadon 50 mcg/ml, Fentanylcitrate, Dechra Veterinary Products Deutschland GmbH) and ketamine (Ketamin 10%, Ketaminhydrochlorid, WDT – Wirtschaftsgenossenschaft Deutscher Tierärzte eG), initially 30 mg/kg metamizole three times daily IV (Metamizol WDT, 500 mg/kg, Metamizol-Natrium, WDT – Wirtschaftsgenossenschaft Deutscher Tierärzte eG), followed by 0.1 mg/kg meloxicam once daily PO (Melosus 1.5 mg/ml, Meloxicam, Cp-Pharma Handelsgesellschaft GmbH) and 10 mg/kg amoxicillin/clavulanic acid two times daily PO (Amoxiclav 40/10 mg, Amoxicillin-Trihydrat, Kalium-Clavulanat) CP-Pharma Handelsgesellschaft GmbH).

The dog was discharged 2 days later with normal micturition. Microbiological examination of the hydrocolpos fluid was negative. At the 2-year follow-up, the patient remained asymptomatic.

## Discussion

Hydrocolpos is a rare congenital malformation in veterinary medicine. It occurs particularly in combination with an imperforate hymen or a missing vestibulovaginal connection (9, 11, 12, 14–18). In total, eight case reports involving 10 dogs have been published (Table 1), describing a spectrum of clinical signs, diagnostic steps, and therapeutic interventions aimed at resolving typical clinical signs such as stranguria, dysuria, and tenesmus (11, 12, 15–17).

**Table 1:** Summary of case reports in veterinary medicine: hydrocolpos.

Case number	Literature	Signalment	Clinical signs	Diagnostics	Diagnosis	Therapy	Clinical course
1	Palm et al. (12)	Chihuahua mix, 15 months old, spayed	Dysuria, stranguria, dyschezia, occasional urinary incontinence	Abdominal ultrasonography, computed tomography, cystourethroscopy, vaginoscopy, fluoroscopy	Congenital imperforate vestibulovaginal connection with secondary hydrocolpos	Initial laparotomy with ovariohysterectomy; after 1.5 months, endoscopic perforation of the vestibulovaginal connection using a guidewire, ballon dilation of the cervical stenosis, enhancement of the opening (2 cm) via laser, rinsing	Initial improvement; after 1 month, recurrence of hydrocolpos; second intervention recommended; euthanasia; no pathohistological examination
2	Palm et al. (12)	Mixed-breed dog, 14 months old, spayed	Strangury, dysuria, urinary incontinence, dyschezia, tenesmus	Abdominal ultrasonography, fine-needle aspiration, cystourethroscopy, vaginoscopy	Congenital imperforate vestibulovaginal connection with secondary hydrocolpos	Endoscopic perforation using biopsy forceps, enhancement of the opening via laser (1,5 cm)	Endoscopic control after 2 weeks (unremarkable); long-term outcome: no clinical signs for 6 years
3	Viehoff and Sjollema (11)	Maltese, 2 years old, intact female	No clinical symptoms; incidental finding during ovariohysterectomy	Vaginoscopy, retrograde vagino-urethrocystography, abdominal sonography	Congenital vaginal obstruction at the level of the vestibulovaginal connection with secondary hydrocolpos (fibrotic connection)	Laparotomy with omentalization (not successful); episiotomy and anastomosis of the caudal vagina and the vestibule	At 24 months postoperatively, the patient remained clinically unremarkable with no recurrence
4	Viehoff and Sjollema (11)	Golden Retriever, 1 year old, intact female	Tenesmus; minimal vaginal discharge during first heat	Abdominal ultrasonography, retrograde vagino-urethrocystography	Congenital complete obstruction between the vestibule and the caudal vagina (vaginal agenesia) with secondary hydrocolpos	Ovariohysterectomy and episiotomy with a tension-free anastomosis of the vagina and the vestibule	At 15 months post- operatively, the patient remained clinically unremarkable
5	Kim et al. (9)	Shih-Tzu, 9 years old, intact female	Abdominal pain, abdominal distension, fever, anorexia, no proestrus bleeding observed since birth, subsequent urinary incontinence	Abdominal radiography, abdominal ultrasonography, explorative laparotomy, vaginoscopy	Persistent hymen with secondary pyocolpos; postoperative recurrent pyocolpos	Ovariohysterectomy and splenectomy; initial hymenotomy; 1 month later, endoscopic dissection of the hymen and lavage	1 month after laparotomy, blunted pyocolpos; development of peritonitis and sepsis after endoscopic dissection of the hymen; death 3 days after surgery
6	Tsumagari et al. (16)	Golden Retriever, 38 months old, intact female	Dysuria, dyschezia	Vaginoscopy, abdominal radiography, abdominal ultrasonography	Imperforate hymen with hydrocolpos and a vaginal polyp	Ovariohysterectomy/partial vaginectomy; dissection of the hymen via episiotomy	3 weeks post- surgery, normal micturition; 20 months after surgery, urinary incontinence
7	Gee et al. (14)	Bullmastiff, 14 months old, female, intact	Seven months of intermittent vaginitis since the first heat, mucopurulent vaginal discharge, vulvar swelling	Abdominal radiography, barium enema, explorative laparotomy	Segmental aplasia of the Müllerian duct system (9 cm obstruction of the cranial vagina) with secondary hydrometra/hydrocolpos	Surgical resection of the aplastic vaginal segment; end-to-end anastomosis of the vagina; intraoperative drainage of the uterus	No postoperative complications; 30 months later, successful gestation (8 puppies); no further complications
8	Alonge et al. (18)	Labrador Retriever, 1 year old, intact female	Vaginal discharge, mild apathy, a single episode of vomiting	Abdominal ultrasonography, explorative laparotomy	Congenital malformation of the tunica muscularis of the cranial vagina with secondary hematopyocolpos (vaginal ectasia due to muscular disorganization)	Ovariohysterectomy and partial vaginectomy with vaginoplasty	No postoperative complications; vaginal discharge few days later; 12 months later, the patient remained clinically unremarkable
9	Blackford et al. (15)	Dachshund, 8 years old, neutered	Acute, painful perineal bulge, dysuria, pollakiuria, tenesmus, dyschezia	Computed tomography, vaginoscopy	Imperforate hymen with secondary hydrocolpos	Perforation of the membrane; endoscopic removal of the remaining hymen	Clinical signs resolved; at the 2-month follow-up, the patient remained clinically unremarkable
10	Kim et al. (17)	Shih Tzu, 5 years old, partially neutered	Initial presentation: 2 days of anorexia and apathy; 50 days post-surgery: dysuria, dyschezia, pollakiuria, and infertility	Abdominal radiography, abdominal ultrasonography, laparotomy	First diagnosis: bilateral ureterolithiasis with hydronephrosis (left > right), pyometra, and azotemia. Second diagnosis: pyocolpos due to an imperforate hymen and vestibulovaginal stenosis	1. First surgery: left-sided nephroureterectomy, right-sided ureterotomy with stent implantation, and hysterectomy. 2. Second surgery (50 days later): Cruciform incision of the hymen, drainage of the pyocolpos, resection of the stenotic area, vestibulovaginal anastomosis via episiotomy, and removal of the ureteral stent	Post-surgery, no clinical signs; normal micturition and defecation; no relapse

These 10 dogs differed greatly in terms of signalment. Dogs of various sizes and breeds, including Chihuahua and Bullmastiff, can be affected by hydrocolpos, regardless of castration status [intact (*n* = 6) (9, 11, 14, 16, 18), spayed (*n* = 2), and neutered (*n* = 2) (12, 15, 17)] and age (1 to 9 years). Most dogs, as in the present case, exhibited micturition disorders (6/10; Table 1), followed by dyschezia (5/10), vaginal discharge (3/10), and tenesmus (2/10). Only one dog showed no clinical signs. The congenital abnormality was an incidental finding during ovariohysterectomy.

In contrast to the present case, all of these dogs developed hydrocolpos due to a congenital anomaly, including an imperforate vestibulovaginal connection (11, 12), vaginal atresia (11), segmental aplasia of the Müllerian duct system (14), congenital malformation of the tunica muscularis (18), or a persistent or imperforate hymen (9, 15–17).

Trauma-induced secondary vaginal atresia resulting in hydrocolpos, as was suspected in the present case, has not been previously described. Pre-existing complete vaginal atresia associated with an imperforate hymen or a missing vestibulovaginal connection seems to be unlikely in our dog because mating was successful. Detailed information regarding possible pre-existing vaginal congenital malformations, such as incomplete vaginal atresia, mating-related injuries, obstetric complications during dystocia, or the course of surgery, was unavailable. Therefore, it remains possible that minor pre-existing anomalies or soft-tissue trauma could have led to secondary vaginal atresia and, consequently, hydrocolpos.

In general, childbirth-related injuries or complications arising from surgical interventions can lead to tissue lacerations followed by fibrotic processes, causing functional or complete stenosis such as secondary vaginal atresia.

In addition, one case report in human medicine describes trauma-induced vaginal atresia and hematocolpos after obstetric injury, supporting this hypothesis (19). The woman had delivered her child by ventouse-assisted delivery and, 5 months after childbirth, developed clinical signs such as abdominal pain, amenorrhea, and urinary retention. Surgical intervention to restore the vagina and opening the hematocolpos led to complete resolution of the clinical signs. Abdominal ultrasonography and vaginoscopy were sufficient to establish the diagnosis in this case.

This does not apply to our case, where ultrasonography revealed a fluid-filled structure of unclear origin.

Further diagnostic techniques, such as radiography with or without contrast agents, fluoroscopy, vaginoscopy, computed tomography, and exploratory laparotomy, are well-described tools for diagnosing hydrocolpos (Table 1) (9, 11, 12, 14–18). Computed tomography, vaginoscopy, and retrograde cystography led to a precise description of the malformation in the present dog.

A minimally invasive surgical procedure was initially chosen because it was suspected that the polyp in the area of the ostium urethrae externum could be the main cause of the micturition disorder and that a minimally invasive intervention would be sufficient to resolve clinical signs. This was supported by retrograde cystourethrography, which demonstrated a dilated urethral lumen proximal to the external urethral ostium, with no evidence of extrinsic urethral compression by the hydrocolpos itself.

The clinical progression strongly supported this initial rationale. After the surgical removal of the polyp and widening of the ostium urethrae externum, the micturition disorder resolved completely and did not recur. However, the subsequent re-presentation of the patient was characterized exclusively by severe tenesmus, perianal swelling, and rectal prolapse.

This shift in clinical signs indicates that the vaginal polyp and the hydrocolpos represented two distinct, albeit anatomically adjacent, mechanical issues. While the polyp caused a focal mechanical urinary outflow obstruction directly at the ostium urethrae externum, the progressively expanding hydrocolpos exerted strict dorsal mechanical pressure on the rectum within the pelvic canal, ultimately triggering severe tenesmus. Urethral patency was not compromised.

A vaginal polyp is a reactive hyperplasia of the myxoid subepithelial zone, which is induced by estrogen. These polyps are well known to cause micturition disorders. In contrast to our case, spaying and surgically removing such polyps usually result in permanent resolution of the clinical signs (20).

The presence of the polyp in our case suggests two plausible pathological mechanisms underlying the overall disease progression. First, the pre-existing polyp may have acted as a mechanical obstruction within the pelvic canal during parturition, directly causing the initial dystocia, which subsequently resulted in birth trauma and secondary vaginal atresia. Alternatively, severe birth-related laceration of the vestibular mucosa surrounding the urethral opening may have stimulated the formation of hyperplastic granulation tissue, resulting in polyp formation and fibrotic occlusion, ultimately leading to hydrocolpos.

Only one case report has described the simultaneous occurrence of a cystic polyp and hydrocolpos in a dog presenting with dysuria and dyschezia (16). In that report, a 38-month-old bitch with a medical history of treated vaginitis and cystic calculi showed an absence of bloody discharge during proestrus. The final diagnosis was achieved via vaginal endoscopy, revealing an intact hymen located cranial to the external urethral orifice alongside a cystic polyp. Curative treatment in that patient required an exploratory laparotomy with ovariohysterectomy, combined with an episiotomy to dissect the hymen (16).

The concurrence of a vaginal polyp and hydrocolpos accounts for the diagnostic and therapeutic complexity of this case.

As documented in the literature, establishing a definitive diagnosis of hydrocolpos can be particularly challenging, even in the absence of a concurrent polyp. Initial misdiagnosis or delayed diagnosis has been frequently reported. For instance, in one case, the condition was initially misdiagnosed as stump pyometra. A re-evaluation performed 2 weeks later due to persistent stranguria and tenesmus finally led to the correct diagnosis of hydrocolpos (12). In another report, a severely distended vagina was noted incidentally during ovariohysterectomy, but the definitive diagnosis of hydrocolpos was only established 4 weeks postoperatively, when the patient was successfully treated by episiotomy (11). Furthermore, a particularly complex case involved an initial nephroureterectomy and hysterectomy performed due to severe pyometra and hydronephrosis. Hydrocolpos was only diagnosed 50 days later when the bitch presented with severe dyschezia and dysuria (17).

Resolution of the clinical signs in the presented case was achieved by surgically emptying the hydrocolpos and creating a stoma between the mucous membrane of the hydrocolpos and the vaginal mucosa via episiotomy. Even 2 years after the procedure, the dog remained asymptomatic.

A review of the literature confirms the assumption that only the drainage of hydrocolpos is an adequate therapeutic intervention (Table 1). In the summarized case reports, successful treatment was achieved by partial vaginectomy, perforation of the hymen, restoration of the vestibulovaginal connection by anastomosis, or hymenal perforation followed by balloon dilatation, with or without episiotomy. Exploratory laparotomy with ovariohysterectomy or omentalization was ineffective (9, 11). In one report, an initial laparotomy with intraoperative vaginal flushing failed to evaluate hymenal patency, leading to recurrence of stump pyocolpos 1 month later. A second endoscopic procedure was therefore required to dissect the persistent hymen and evacuate the fluid. However, the patient ultimately succumbed to sepsis 3 days after the procedure (9). Regarding long-term outcomes, only one patient developed urinary incontinence 20 months postoperatively (16). Another patient treated with an end-to-end vaginal anastomosis achieved a successful pregnancy and delivered eight healthy puppies 30 months later (14).

A main limitation of this case report is the limited information available regarding the medical history of the dog. Nothing is known about the presence of potentially less severe congenital vaginal malformations prior to mating or the dog's micturition behavior before or immediately after dystocia. From the first day after arrival in Germany, the dog exhibited micturition disorders. In comparison to the human case, micturition disorders occurred in nearly the same time frame after birth: five months after childbirth in the human case (19) and approximately six months after dystocia in the presented case.

Although the definitive pathogenesis of the vaginal atresia and resulting hydrocolpos could not be determined, this case shows that hydrocolpos should be considered a differential diagnosis in dogs with persistent stranguria, tenesmus, and dyschezia after obstetric complications. A combination of imaging tools can facilitate the diagnosis. In the long term, invasive surgical treatment represents the most promising treatment option. The dog presented in this case remained asymptomatic 2 years after the procedure.

## Conclusion

To the best of the authors' knowledge, this is the first case report describing suspected secondary, traumatically induced vaginal atresia and hydrocolpos. Drainage of the hydrocolpos by creating a stoma between the hydrocolpos and the vaginal canal via episiotomy was an effective treatment in the presented case.

## Data Availability

The original contributions presented in the study are included in the article/supplementary material, further inquiries can be directed to the corresponding author.
